# Mastication and Specialization

**Published:** 1907-03

**Authors:** A. P. Gordy

**Affiliations:** Columbus, Ga.


### MASTICATION AND SPECIALIZATION.

By Dr. A. P. Gordy, Columbus, Ga.

Some time ago Professor William Osler delivered a lecture before the students of the Royal Dental Hospital in London, and among the many interesting things, the learned doctor laid special stress upon the importance of proper mastication. His remarks attracted attention, not only from the medical and dental profession, but many newspapers commented editorially, as though some new-born truth had been launched by this remarkable man; when, as a matter of fact, advanced dental thinkers have, single-handed alone, taught for years that without proper assimilation, good health is impossible; without proper digestion, assimilation is imperfect; and as digestion begins in the mouth, it is a physical impossibility for one whose mouth is not in a normal condition and who does not masticate properly to have that perfect health one should enjoy.

This subject of thorough mastication has been the subject of much ridicule. Many seem to think it is a crazy notion of some men and do not realize not only the importance of thorough mastication of foods, but also the necessity of the selection of such foods as will require thorough mastication in order to liquify them. Such chewing not only develops the teeth and jaws, but stimulates the salivary glands as well, without which exercise the jaws become contracted, the glands become inactive and do not secrete digestive juices as they should, and indigestion is sure to follow. The primitive notion has been exploded that the mouth was simply an opening through which food is thrust into the stomach to be digested. The greater part of digestion is done in the mouth and indigestion is caused from improper mastication and lack of juices from the salivary glands, due to want of exercise and proper stimulation. When a limb is inactive for six months the muscles waste, and so it is with these glands.

I do not believe that nature intended that things should be put into the baby's mouth to steal away its digestion, and were children required to eat bread crusts and other foods that require chewing in order to be swallowed, and quit loading their delicate stomachs with candies and sweets, the mortality from detention would decrease as though some great specific had been discovered, and the paregoric doctor would be a thing of the past.

It has been demonstrated that when food is thoroughly chewed and saturated with saliva and practically swallows itself, a lesser amount is required to satisfy the appetite and demands of nutrition. The food then passes into the stomach so well prepared as to throw no extra work upon these digestive organs, and they not being over-taxed, perform their functions perfectly. Disregard these laws of nature and the man whose digestive organs are weak must suffer. He is thoroughly transformed from the pleasant, affable man into a disagreeable, disgruntled fault-finder. He is not himself; his disposition is changed; he has bolted his food, succumbed to the fickle fancy of an abnormal appetite, and the road to dyspepsia is short.

We see the maiden upon the threshold of young womanhood, upon whose cheek nature has stamped the ruddy glow of youth and health. Her social career is often brief, and late hours are not uncommon. Waldorf salads, sweets and other uneatables are used daily, and in the rush of social swim, mastication is neglected, the food is bolted, and even ''tired nature's sweet restorer, balmy sleep,'' is almost forgotten. After a few short years the luster does not linger in her eyes, the roses no longer chase the lilies from her cheeks, and then it is that the victim is liable to contract what Fra Elbertus is pleased to call the "Beecham habit."

Says he, in a recent number of a popular magazine, "I see portrayed with all the seductive skill of an expert illustrator, a beautiful young woman with her hair neatly braided down her back. She is arrayed in a beautiful gown that is a dream. Like the Goddess of Liberty in New York harbor, she holds aloft a lighted candle in one hand and in the other—a pill. If the scale of drawing is correct, this pill is about the size of a baseball. The import of the picture is that the beauty is about to swallow the baseball. Beneath the picture is the legend, 'My complexion is perfect because I take one Billson's Bully Boluses every night on retiring.' "

Says Elbertus, "Good money is invested in Fig Syrup, Little Liver Pills and Baseball Boluses in order to have a good complexion and sweet thoughts." Very many people believe this, and the habit begins by gently dallying with the Lady Webster Dinner Pill. It grows, it grows. One pill is sufficient at first, but two are soon required where only one would suffice before; then three are demanded, and soon a change is required from pills to Fig Syrup, then Mother Shipley's Tea, and back to the pills; from Carter's to Pierce's, then Ayers' to Beecham's Bulsoms, and at last a frantic dash is made for Ripun Tablets. One's stomach has been stored with such a miscellaneous cargo that nature stops, perplexed. Carter is consulted. "She starts! She moves! She seems to feel the thrill of life along her keel." Then come cold chills, hot, bearing, stomach protest; liver lags, kidneys kick, bile accumlates and nature pipes all hands to pump the ship. The patient goes into dry dock and, specialists being consulted, he is told he has a cancer of the stomach, fistula, tuberculosis of the bowels and Bright's disease—and he *has*, or *thinks* he has, which is just as bad.

Now, my friends, I am not complaining. I do not believe in being on the dark side of the street, when just across the way the sun is always shining. I believe in the efficacy of medicine when intelligently administered, and I am in thorough sympathy with that vast array of scientific men who are ever moving onward and upward, and who with conscience as their guide are ever ready to follow where duty calls. Still, there are few men who can approach the versatility of genius which characterized Hypocrates and Galen, and while there are many deformities, defects and pathological conditions which are beyond the reach of the ordinary physician's skill, there are some who treat any disease to which the human family is heir. I do not believe that a man who treats pulpitis, peritonitis, laryngitis or appendicitis can deliver the goods. It is this man who poultices or stabs the victim's cheek for alveolar abscess and leaves a scar as a monument to his ignorance; or who operates for appendicitis, when, lo! he finds that the trouble is in the "table of contents" and not in the appendix at all.

I would not usurp the prerogatives of the medical man, but this is an age of specialism. The oral cavity, its functions and all there is in it, is the dentist's field and an intelligent public will accord us the right to attain the best scientific knowledge possible. It is to the specialist that we look for the most valuable contribution to medical and dental science, and out of this wave of eating-reform preached so long by dentists there has resulted a campaign for proper eating and mastication which has enlisted the aid of distinguished people throughout the world. If the simple life, simple living and simple eating, had been taught years ago to those who now lead the strenuous life, we would not now be called a nation of dyspepetics.

The most wonderful strides in medical and dental science have been in the direction of prevention, and now that the medical world is beginning to lend its mighty influence, why cannot many of the ills imposed on us by Adam and Eve in beautiful Eden be eradicated!

Dental scientists have taken the initiative in many things, and since dentistry was born, over one-half century ago, it has kept time and perfect step to the march of progress and its members have contributed liberally to the healing art. It was in its infancy when Horace Wells, a dentist of Hartford, Conn., discovered nitrous oxide gas and gave to the world the first anæsthetic, "to lull all pain and bring forgetfulness of every sorrow."

Professor Miller, an American dentist residing in Berlin, was first to contribute to science the cause of dental caries.

Professor G. V. Black, of Chicago, one of the world's greatest dental authorities, has contributed more than any living man to the pathology, histology and bacteriology of the mouth.

Dr. J. M. Riggs, a dentist, was the first to call attention to the ravages of a disease which was playing havoc with the gums and teeth of his patients, thus making mastication impossible; and while only in the last few years has it received the attention its gravity merited, it now occupies the time and talent of some of the most eminent dental pathologists in America and throughout the world.

Doctors Garretson, Brophy, Cryer and their colleagues revolutionized the practice of oral surgery, and made it a specialty of dentistry; and "Their method of operative procedure," says the lamented Chapple, "has not been supplanted even in this marvelous age of progress in general surgery."

Doctors Farrar, Kingsley, Angle and others have by their ingenuity and skill made it possible to transform the deformed physiognomy into the face of most perfect mould.

Dentistry, though young, has made more rapid strides than any other specialty of medicine. Science has robbed the dental chair of most of its horrors. The dental instrument is no longer called "the instrument of torture," and in this progressive age "when eager eyes are watching every sail, we cannot dream of the victories that may lie in the future. We do not know what scientific thought might leap from the brain of men," but I do know that we will never live to see the day when teeth will not decay; when bald heads will be covered with hair in a single night; when good health will be more contagious than disease; when microbes and maladies will be driven from the face of the earth; when men and women will not grow old, and when sin itself will be no more.

Still, let us hope that the day is not far distant when an intelligent public will realize the importance of thorough mastication, and when men will specialize for the good of humanity and with a right good will.

